# Analytical and Numerical Investigation of Star Polymers in Confined Geometries

**DOI:** 10.3390/ijms25179561

**Published:** 2024-09-03

**Authors:** Zoriana Danel, Joanna Halun, Pawel Karbowniczek

**Affiliations:** 1 Faculty of Materials Engineering and Physics, Cracow University of Technology, 30-719 Cracow, Poland; pkarbowniczek@pk.edu.pl; 2 Institute of Nuclear Physics, Polish Academy of Sciences, 30-719 Cracow, Poland

**Keywords:** critical phenomena, soft matter, surface effects, polymers, field theory

## Abstract

The analysis of the impact of the star polymer topology on depletion interaction potentials, depletion forces, and monomer density profiles is carried out analytically using field theory methods and techniques as well as molecular dynamic simulations. The dimensionless depletion interaction potentials and the dimensionless depletion forces for a dilute solution of ideal star polymers with three and five legs (arms) in a Θ-solvent confined in a slit between two parallel walls with repulsive surfaces and for the case where one of the surfaces is repulsive and the other inert are obtained. Furthermore, the dimensionless layer monomer density profiles for ideal star polymers with an odd number ($\tilde{f}$ = 3, 5) of arms immersed in a dilute solution of big colloidal particles with different adsorbing or repelling properties in respect of polymers are calculated, bearing in mind the Derjaguin approximation. Molecular dynamic simulations of a dilute solution of star-shaped polymers in a good solvent with N = 901 (3 × 300 + 1 -star polymer with three arms) and 1501 (5 × 300 + 1 -star polymer with five arms) beads accordingly confined in a slit with different boundary conditions are performed, and the results of the monomer density profiles for the above-mentioned cases are obtained. The numerical calculation of the radius of gyration for star polymers with $\tilde{f}$ = 3, 5 arms and the ratio of the perpendicular to parallel components of the radius of gyration with respect to the wall orientation for the above-mentioned cases is performed. The obtained analytical and numerical results for star polymers with an odd number ($\tilde{f}$ = 3, 5) of arms are compared with our previous results for linear polymers in confined geometries. The acquired results show that a dilute solution of star polymer chains can be applied in the production of new functional materials, because the behavior of these solutions is strictly correlated with the topology of polymers and also with the nature and geometry of confined surfaces. The above-mentioned properties can find extensive practical application in materials engineering, as well as in biotechnology and medicine for drug and gene transmission.

## 1. Introduction

New developments in controlled polymerization techniques have made it possible to synthesize complex polymer architectures with controlled dimensions and functionality. One example of such polymers with complex architectures are star polymers, which belong to the wider class of branched polymers. It is worth mentioning that star polymers have been synthesized and characterized since 1980. For the past ten years or so, star polymers and dendritic polymers (dendrimers) have been widely used in biomedical applications such as drug delivery, tissue engineering, gene delivery, diagnostics, and antibacterial biomaterials [1]. It is essential to thoroughly comprehend the statistical and conformation properties of star polymers, since such understanding is connected with the investigation of micellar and other polymeric surfactant systems [2,3] as well as networks [4,5]. Additionally, star polymers can be widely applied in the production of new-generation functional materials and can be used in nano-technology and biomedical sciences as drug, gene, and siRNA/DNA vectors [1]. Recently, promising results in the synthesis of immobilized enzymes in organic–inorganic hybrid nanoflowers with greatly improved catalytic activity and stability were obtained in [6].

It is no wonder that star polymers have been gaining increasing interest, as they have unique topological structures and attractive physical as well as chemical properties, such as low viscosity in dilute solutions, enhanced stimulus responsiveness, internal and peripheral functionality, and encapsulation capability [1]. As it was shown recently [7], the shape effects of polymer nanoparticles should be taken into consideration when the in vitro and in vivo behaviors of such particles are discussed. For example, spherical polymer nanoparticles had longer blood circulation time, higher tumor accumulation, and a greater ability to extravasate from tumor vessels than cylindrical polymer brushes [7].

Star polymers have a three-dimensional hyperbranched structure and are formed from linear polymers (star arms) of the same or different molecular weight which radiate out from a central core. These macromolecules can be classified according to the monomer composition, chemical structure, sequence distribution of the arms, and molecular nature of the core [1]. A vast number of star polymer structures can be obtained through controlled polymerization. In this respect, it is essential to consider star polymer structures with block copolymers, network–core, and end-functionalized star polymers. In general, the synthesis of star polymers can be generated by arm (leg)-first, core-first, and grafting-onto approaches. Each of these methods has a distinct set of advantages and disadvantages, which have recently been discussed in [1]. It is worth mentioning that the arm-first method assumes the use of a multifunctional termination agent or a cross-linking of linear polymers prepared using living-controlled polymerization techniques [1]. Star polymers, due to their exclusive structure, exhibit some remarkable properties non-existent in simple linear polymers. On the other hand, it is interesting to analyze the behavior of a dilute solution of star polymers immersed in confined geometries, like a slit between two parallel walls, or of star polymers plunged in a solution of colloidal particles of a large but finite size and with different adsorbing as well as repelling properties with respect to the polymers.

As was mentioned in [8], we can differentiate two cases which generally lead to qualitatively different effects during the investigation of microscopic interactions in polymer–colloid mixtures. One of them leads to the protection of the colloidal particles from flocculation [9,10], and the other one leads to the depletion effect [11], i.e., when polymers are expelled from the region between two particles due to entropic reasons. In such a situation, the depletion interaction potential gives rise to the depletion force between colloidal particles or nanoparticles and a surface [8]. It can be assumed that the magnitude of the depletion force depends on the concentration of the polymer solutions, the topology and effective size of the polymers, the size and shape of the colloidal particles or nanoparticles, and the separation distance. Improvements in experimental techniques have made it possible to measure with high accuracy the depletion force between a wall and a single colloidal particle immersed in a dilute solution of nonionic linear polymers in a good solvent [11,12,13].

The universal density–force relation proposed some time ago in [14] for the different cases of a dilute solution of linear polymers in confined geometries as well as for the case of a semi-dilute solution of free linear polymers in a semi-infinite space containing a mesoscopic colloidal particle of arbitrary shape was corroborated in [15] and verified using numerical methods in [16,17] for the case of two repulsive walls.

In a series of our recent papers [18,19], the density–force relation for a dilute solution of linear ideal and real polymers with the EVI confined in a slit geometry of two parallel walls with different boundary conditions as well as for the case of a dilute solution of linear polymers confined in a half space containing a spherical colloidal particle of big radius was investigated by analogy, as was proposed in [15], and the corresponding universal amplitude ratio was obtained in the framework of the massive field theory approach directly in d=3 dimensions up to the one-loop order. Additionally, the interaction of long, flexible, non-adsorbing linear polymers with big and small colloidal particles of mesoscopic size with different shape was the subject of a series of papers [20,21].

In order to comprehend the significance of the polymers’ topology, we should note that the investigation of the physical effects arising from the confinement of polymers and their topology plays an important role in the shaping of individual chromosomes and in the process of their segregation, as was shown in [22]. The computational investigation of the good solvent solution properties of knotted rings with minimal crossing number in the range between $m_{c}=0$ and $m_{c}=9$ as well as for the case of star polymers with number of arms in the region between f=2 and f=20 were carried out by combining the MD simulation technique and path-integral calculations in [23]. Furthermore, numerical calculations performed in [23] suggested that the configurational properties of knotted rings and star polymers in a good solvent show a similar decrease with increasing minimal crossing number and number of star polymer arms. Thus, it is very interesting to compare the analytical results of the statistical properties of ring and star polymers.

In a series of our recent papers [8,24,25,26], an investigation into the influence of the topology of ring polymers on the depletion interaction potential and the depletion force was performed. The obtained results indicate that a dilute solution of ring polymers behaves in a completely different way than a dilute solution of linear polymers in confined geometries. The explanation of these results for the depletion interaction potential and the depletion force can be derived from the assumption that topological effects in this situation start to play a vital role.

Unfortunately, the analytical understanding of the processes which take place in the case of immersing a dilute solution of star polymers with an odd number $\tilde{f}=3,5$ of arms in confined geometries like a slit of two parallel walls or in a solution of colloidal particles of mesoscopic size with different boundary conditions is still incomplete, and extensive investigation is required, especially in the context of the dimensionless monomer density profiles, the depletion interaction potentials, and the depletion forces. Consequently, the above-mentioned research is a subject of the analytical and numerical investigation in the present paper. The obtained analytical and numerical results for star polymers with an odd number $\tilde{f}=3,5$ of arms are compared with our previous results for linear polymers and ring polymers in confined geometries.

## 2. The Method

### 2.1. The Model and the Polymer–Magnet Analogy

In our investigations, we consider a dilute solution of star flexible polymers with an odd number $\tilde{f}=3,5$ of arms confined in a slit of two parallel walls with repulsive surfaces as well as the case of walls with mixed surfaces, when one surface is repulsive and the other is inert. We allow for the exchange of polymer coils between the slit and the reservoir outside the slit. We consider a sufficiently dilute polymer solution, and thus the interchain interactions and the overlapping between different polymers and different arms of star polymers can be neglected, and in accordance with that, it is sufficient to consider the behavior of a single star polymer with a different number $\tilde{f}=3,5$ of arms in confined geometry. In general, the behavior of a single ideal star polymer at Θ-solvent can be described using the model of random walk (RW), and the behavior of a real star polymer with the excluded volume interaction (EVI) for the temperatures above the Θ—point can be described using the model of self-avoiding walk (SAW). When the EVI between monomers becomes relevant, the star polymer coils are less compact than in the case of ideal star polymers. The situation when the solvent temperature is below the Θ—temperature corresponds to a poor solvent where polymer coils tend to collapse [27,28].

Bearing in mind the well-known similarity between the statistics of long flexible polymers and the critical behavior of magnetic systems developed some time ago by de Gennes [29], we can use powerful field theory methods and techniques for the investigation of the critical behavior of star polymers. Thus, the scaling properties of long-flexible polymer chains in the limit of an infinite number of monomers *N* may be derived from a formal n→0 limit of the field-theoretical $\phi^{4}$O(n)—vector model at its critical point [29]. The value 1/N in this model plays the role of a critical parameter analogous to the reduced critical temperature $\tau =\frac{T-T_{c}}{T_{c}}$, where $T_{c}$ is the critical temperature in the magnetic systems. In order to better understand the meaning of the value 1/N, we can present the connection of this value with the bulk correlation length ξ. As is known [29], the bulk correlation length ξ, which behaves near criticality in the magnetic systems, can be expressed as:

(1) $$\xi ∼{\left|\tau \right|}^{-\nu }$$

On the other hand, in a dilute solution of polymers, scales like the average end-to-end distance:

(2) $$\xi ∼\sqrt{<R^{2}>}∼N^{\nu },$$

where ν is a critical exponent which, in the case of a dilute polymer solution, corresponds to a Flory critical exponent. The critical exponent ν equals 1/2 at d=3 dimensions in the case of ideal polymers and ν≈0.588 for real polymer chains with the EVI.

Moreover, as it was mentioned earlier by de Gennes [30,31] and Barber et al. [32], it is possible to observe a formal analogy between the adsorption of polymers on surfaces and the critical behavior of a magnet with a free surface. The deviation from the adsorption threshold $\left(c\propto \left(T-T_{a}\right)/T_{a}\right)$ when polymers start to adsorb on the surface ($T_{a}$ is the adsorption temperature) changes sign at the transition between the adsorbed (the so-called normal transition, when c<0) and the non-adsorbed state (ordinary transition, when c>0) [33,34,35]. In accordance with that, the value *c*, which corresponds to the adsorption energy divided by $k_{B}T$ (or the surface enhancement constant in the field theoretical treatment), plays the role of a second critical parameter. Thus, the adsorption threshold for long-flexible star polymers, where 1/N→0 and c→0, corresponds to a multicritical phenomenon.

When a dilute polymer solution is confined to a slit of two parallel walls, the properties of the system depend on the ratio L/ξ as was shown in [36], where *L* is the distance between two walls.

As it was noticed earlier by de Gennes [29], the partition function $Z(x,x^{\prime })$ of a single linear polymer chain with two ends fixed at x and $x^{\prime }$ is connected with the two-point correlation function $G^{\left(2\right)}\left(x,x^{\prime }\right)=<\vec{\phi }\left(x\right)\vec{\phi }\left(x^{\prime }\right)>$ in the framework of the $\phi^{4}$O(n)—vector model for *n*-vector field $\vec{\phi }\left(x\right)$ with the components $\phi^{i}\left(x\right)$, i=1,…,n (and x=(r,z)) via the inverse Laplace transform $\mu_{0}^{2}\to L_{0}$:

(3) $$Z\left(x,x^{\prime };N,v_{0}\right)={\mathrm{IL}}_{\mu_{0}^{2}\to L_{0}}\left(<\vec{\phi }\left(x\right)\vec{\phi }\left(x^{\prime }\right)>{|}_{n\to 0}\right)$$

in the limit, where the number of *n* components tends to zero and $v_{0}$ is the bare coupling constant which characterizes the strength of the EVI in the case of a dilute solution of real polymers in a good solvent. In the case of a dilute solution of ideal polymers at Θ—solvent, the bare coupling constant $v_{0}$ equals zero. The conjugate Laplace variable $L_{0}$ has the dimension of length squared. Moreover, $L_{0}$ is proportional to the total number of monomers *N* and equals $R_{g}^{2}=R_{x}^{2}/2$ for an ideal linear polymer in the bulk.

On the other hand, the Laplace transformed function $G^{\left(2\right)}\left(x,x^{\prime }\right)$ can be expressed as the n→0 limit of the functional integral over vector fields $\vec{\phi }\left(x\right)$ with *n* components $\phi^{i}\left(x\right)$, i=1,…,n and x=(r,z):

(4) $$G^{\left(2\right)}\left(x,x^{\prime }\right)=\int D\left[\vec{\phi }\left(x\right)\right]e^{-H[\vec{\phi }]},$$

with the Landau–Ginzburg–Wilson (LGW) Hamiltonian $H(\vec{\phi })$ describing the system in a semi-infinite (j=1) [37] or confined geometry of two parallel walls (j=1,2) [36]. The fundamental two-point correlation function of the free theory corresponding to the effective LGW Hamiltonian in a mixed momentum-space (p,z) representation is:

(5) $$G_{ij}^{\left(2\right)}\left(p,p^{\prime };z,z^{\prime }\right)={\left(2\pi \right)}^{d-1}\delta_{ij}\delta \left(p+p^{\prime }\right)\;{\tilde{G}}_{\|}\left(p;z,z^{\prime };\mu_{0},c_{1_{0}},c_{2_{0}},L\right)\;,$$

where the free Gaussian propagator ${\tilde{G}}_{\|}\left(p;z,z^{\prime };\mu_{0},c_{1_{0}},c_{2_{0}},L\right)$ of the model in p−z representation was obtained in one of our previous papers [36].

In the case of a dilute solution of star polymers with a different number $\tilde{f}=3,5$ of arms in a Θ-solvent immersed in a confined geometry like a slit of two parallel walls, the respective correlation function should be modified by analogy, as was proposed by [3,4,5] for the case of infinite space and semi-infinite space [38], and can be written in the form:

(6) $$G_{st}^{\tilde{f}}\left(\mu_{0}\right)=<\sum_{j_{1},\ldots j_{\tilde{f}}=1}^{n}T_{i_{1},\ldots ,i_{\tilde{f}}}\phi^{i_{1}}\left(x_{0}\right)\ldots \phi^{i_{\tilde{f}}}\left(x_{0}\right)\phi^{j_{1}}\left(x_{1}\right)\ldots \phi^{j_{\tilde{f}}}\left(x_{\tilde{f}}\right){>}_{n\to 0}^{H_{st}\left[\vec{\phi }\right]},$$

where the average <…> in Equation (6) is understood with respect to the Hamiltonian $H_{st}\left[\vec{\phi }\right]$ of the system:

(7) $$\begin{array}{ccc} H_{st}\left[\vec{\phi }\right] & = & \sum_{a=1}^{\tilde{f}}\int d^{d}x\left\{\frac{1}{2}{\left(\nabla {\vec{\phi }}_{a}\right)}^{2}+\frac{\mu_{0,a}^{2}}{2}{{\vec{\phi }}_{a}}^{2}\right\}\\ & + & \sum_{a=1}^{\tilde{f}}\sum_{j=1}^{2}\frac{c_{j_{0},a}}{2}\int d^{d-1}r{{\vec{\phi }}_{a}}^{2}, \end{array}$$

where $\mu_{0,a}^{2}$ is the so-called “bare mass” in field-theoretical treatment, which, in the case of a magnet, corresponds to the reduced temperature:

(8) $$\mu_{0,a}^{2}-\mu_{0c,a}^{2}∼\tau ,$$

where $\mu_{0c,a}^{2}$ is its critical value. The vector fields ${\vec{\phi }}_{a}$, $a=1,\ldots ,\tilde{f}$ have n components $\phi_{a}^{i}$, i=1,…,n. The correspondent partition function for the star polymer can be obtained via the inverse Laplace transform of the correlation function in Equation (6) by analogy, as was mentioned in [3,4,5] for infinite space and in [38] for semi-infinite space.

We consider a dilute solution of star polymers with an odd number $\tilde{f}=3,5$ of arms immersed into a slit of two parallel walls and take into account that in field theory, the star vertex is related to the local composite operator (see [39]) appearing in Equation (6):

(9) $${\left(\phi \right)}_{st}^{\tilde{f}}\left(x\right)=T_{i_{1},\ldots ,i_{\tilde{f}}}\phi^{i_{1}}\left(x\right)\ldots \phi^{i_{\tilde{f}}}\left(x\right),$$

where $T_{i_{1},\ldots ,i_{\tilde{f}}}$ is a traceless symmetric SO(*n*) tensor satisfying the condition:

(10) $$\sum_{i=1}^{n}T_{i,i,i_{3},\ldots ,i_{\tilde{f}}}=0.$$

In our consideration, we assume that the walls in a slit are located at the distance *L* one from another in the *z*-direction such that the surface of the bottom wall is located at z=0 and the surface of the upper wall is located at z=L. The surfaces of the system are characterized by a certain surface enhancement constant $c_{j_{0},a}$, where j=1,2, and *a* can change from a=1 up to $a=\tilde{f}$.

The interaction between star polymers with a different number of arms $\tilde{f}=3,5$ and the surfaces of the walls is implemented by different boundary conditions. In the case of two walls with repulsive surfaces, the Dirichlet–Dirichlet boundary conditions (D-D b.c.) can be written in the form:

(11) $${\vec{\phi }}_{a}\left(r,0\right)={\vec{\phi }}_{a}\left(r,L\right)=0\;or\;c_{1}\to +\infty ,\;c_{2}\to +\infty ,$$

and for the mixed case of one repulsive and the other one inert surface, the Dirichlet–Neumann boundary conditions(D-N b.c.) are:

(12) $${\vec{\phi }}_{a}\left(r,0\right)=0,\;\frac{\partial {\vec{\phi }}_{a}\left(r,z\right)}{\partial z}{|}_{z=L}=0\;or\;c_{1}\to +\infty ,\;c_{2}=0.$$

The most common parameter to denote the polymer chain size that is observable in experiments is the radius of gyration $R_{g}$. For example, for linear polymer chains: $<R_{g}^{2}>\;=\chi_{d}^{2}\frac{<R_{x}^{2}>}{2}$, where $\chi_{d}$ is a universal numerical prefactor depending on the dimensionality *d* of the system (see Refs. [35,40,41]) and $R_{x}$ is the projection of the end to end distance R onto the direction of the *x* axis. In the case of ideal polymers, one has $\chi_{d}^{2}=\frac{d}{3}$. Moreover, the mean square radius of gyration $R_{g,\tilde{f}}$ for star polymer chains is [42]:

(13) $$<R_{g,\tilde{f}}^{2}>=\frac{Nl^{2}}{6\tilde{f}}\left(3-\frac{2}{\tilde{f}}\right),$$

and can be rewritten for $\tilde{f}=3,5$ arms in the form, respectively:

(14) $$<R_{g,\tilde{f}=3}^{2}>\to \frac{7}{18}<R_{x}^{2}>,$$

(15) $$<R_{g,\tilde{f}=5}^{2}>\to \frac{13}{50}<R_{x}^{2}>,$$

where *l* is the monomer size. Taking into account the Derjaguin approximation [43] offers us the possibility to investigate the interaction of star polymers with big mesoscopic colloidal particles and show how methods of field theory with boundaries [34,36,37] allow us to explain basic properties of polymer–colloid mixtures and polymer-induced interactions between the particles. In the case of ideal chains, integrating out the polymer degrees of freedom is a nontrivial task in the presence of the colloidal particles, as was mentioned some time ago by Eisenriegler [44]. Moreover, the application of field theory methods and techniques is very useful for the investigation of the behavior of a dilute solution of star polymers with an odd number $\tilde{f}=3,5$ of arms at Θ temperature where coil–globule transition takes place and in the case of a good solvent in confined geometries of two parallel walls with different adsorbing or repelling properties in respect to the polymers.

### 2.2. Thermodynamic Description

We consider a situation when a dilute solution of ideal star polymers with an odd number $\tilde{f}=3,5$ of arms in a slit is in equilibrium contact with an equivalent solution in the reservoir outside the slit. In the present investigation of a dilute solution of star polymers, we modify the thermodynamic description previously proposed for the case of linear polymers in [36,45].

As was shown in [45], the free energy of the interaction between the walls in a grand canonical ensemble is defined as the difference of the free energy of an ensemble where the separation of the walls is fixed at a finite distance *L* and where the walls are separated infinitely far from each other:

(16) $$\delta F_{\tilde{f}}=-k_{B}T\;\tilde{N}\;\ln\left(\frac{Z_{\tilde{f},\|}\left(L\right)}{Z_{\tilde{f},\|}\left(L\to \infty \right)}\right)\;=\;-k_{B}T\;\tilde{N}\;\left\{\ln\left(\frac{Z_{\tilde{f},\|}\left(L\right)}{Z}\right)-\ln\left(\frac{Z_{\tilde{f},\|}\left(L\to \infty \right)}{Z}\right)\right\},$$

where $\tilde{N}$ is the total amount of star polymers in the solution and *T* is the temperature. It should be mentioned that here the value $Z_{\tilde{f},\|}\left(L\right)$ is the partition function of one star polymer located in volume *V* containing two walls at a distance *L*. In the case of star polymers with an odd number $\tilde{f}=3,5$ of arms, all equations for the correspondent partition functions, the free energy, the depletion interaction potentials, and the depletion forces should be modified because we have to do with polymers where a different number of arms are connected in the core. For the sake of convenience, we renormalized the partition functions $Z_{\tilde{f},\|}\left(L\right)$ and $Z_{\tilde{f},\|}\left(L\to \infty \right)$ on the function $Z=V{\hat{Z}}_{b}$, where ${\hat{Z}}_{b}={\mathrm{IL}}_{\mu_{0}^{2}L^{2}->\frac{7R_{x}^{2}}{18L^{2}}}\left[\frac{1}{\mu_{0}^{6}L^{6}}\right]$ for a star polymer with $\tilde{f}=3$ arms and ${\hat{Z}}_{b}={\mathrm{IL}}_{\mu_{0}^{2}L^{2}->\frac{13R_{x}^{2}}{50L^{2}}}\left[\frac{1}{\mu_{0}^{10}L^{10}}\right]$ for a star with $\tilde{f}=5$ arms in order to obtain dimensionless expressions for the respective scaling functions. As in [36,45], we split the total volume of the system *V* into two independent subsystems inside ($V_{i}$) and outside ($V_{o}$) the slit.

The respective reduced free energy of interaction $\delta f_{\tilde{f}}$ per unit area A=1 for the case of star polymers confined in a slit of two parallel walls can be obtained. After performing Fourier transform in the direction parallel to the surfaces and integration over $d^{d-1}r$ in Equation (16), the functions ${\hat{Z}}_{i,\tilde{f}}$ and ${\hat{Z}}_{HS_{i},\tilde{f}}$ depend only on the *z*-coordinates perpendicular to the walls and we obtain:

$$\begin{array}{ccc} \delta f_{\tilde{f}}=\frac{\delta F_{\tilde{f}}}{n_{B}k_{B}T} & = & \end{array}$$

(17) $$\begin{array}{ccc} L-\int_{0}^{L}dz\frac{{\hat{Z}}_{i,\tilde{f}}\left(z\right)}{{\hat{Z}}_{b}}+\int_{0}^{\infty }dz\left(\frac{{\hat{Z}}_{HS_{1},\tilde{f}}\left(z\right)}{{\hat{Z}}_{b}}-1\right) & + & \int_{0}^{\infty }dz\left(\frac{{\hat{Z}}_{HS_{2},\tilde{f}}\left(z\right)}{{\hat{Z}}_{b}}-1\right). \end{array}$$

The value $n_{B}=\tilde{N}/V$ is the number density of polymer chains in the bulk solution, the function ${\hat{Z}}_{i,\tilde{f}}\left(z\right)$ denoting the partition function of star polymers inside the slit, and ${\hat{Z}}_{HS_{i,\tilde{f}}}\left(z\right)$ with i=1,2 denotes the correspondent partition functions of the star polymer in a half space.

The reduced free energy of interaction $\delta f_{\tilde{f}}$, according to Equation (17), is a function of the dimension of length. Dividing it by another relevant length scale, for example, the size of the chain in bulk, e.g., $R_{x}$, yields a universal dimensionless scaling function of the depletion interaction potential:

(18) $$\Theta_{\tilde{f}}\left(y\right)=\;\frac{\delta f_{\tilde{f}}}{R_{x}}\;,$$

where $y=L/R_{x}$ is a dimensionless scaling variable. The resulting scaling function of the depletion force between two walls induced by the polymer solution is denoted as:

(19) $$\Gamma_{\tilde{f}}\left(y\right)=\;-\frac{d(\delta f_{\tilde{f}})}{dL}\;=\;-\frac{d\Theta_{\tilde{f}}\left(y\right)}{dy}\;.\;$$

According to Equations (16) and (17), in the thermodynamic limit $\tilde{N},V\to \infty$, the total grand canonical free energy $\Omega_{\tilde{f}}$ of the polymer solution of star polymers within the slit is:

(20) $$\Omega_{\tilde{f}}=-n_{B}\;k_{B}\;T\;A\;L\omega_{\tilde{f}}$$

with

(21) $$\omega_{\tilde{f}}\;=\frac{1}{L}\int_{0}^{L}\;dz\;\frac{{\hat{Z}}_{i,\tilde{f}}\left(z\right)}{{\hat{Z}}_{b}}.$$

Taking into account Equations (17) and (20), we can write for unit surface area A=1:

(22) $$\frac{\Omega_{\tilde{f}}}{n_{B}k_{B}T}=f_{b}\;L\;+\;f_{s_{1},\tilde{f}}\;+\;f_{s_{2},\tilde{f}}\;+\;\delta f_{\tilde{f}}\;,$$

with the reduced bulk free energy per unit volume $f_{b}=-1$ and the reduced surface free energy per unit area

(23) $$f_{s_{i},\tilde{f}}=\int_{0}^{\infty }dz\left(1-\frac{{\hat{Z}}_{HS_{i},\tilde{f}}\left(z\right)}{{\hat{Z}}_{b}}\right).$$

## 3. Results

### 3.1. Results of the Depletion Interaction Potentials and the Depletion Force Calculations for Star Polymers with an Odd Number $\tilde{f}=3,5$ of Arms

Let us consider at the beginning the case of a dilute solution of star polymer chains with an odd number $\tilde{f}=3,5$ of arms under a Θ-solvent condition trapped in the slit of two parallel repulsive walls situated at a distance *L* one from another. After the introduction of the respective modifications of the calculation scheme proposed for linear polymers in [36], for the case of star polymer chains with an odd number $\tilde{f}=3,5$ of arms, we obtain the results for the dimensionless depletion interaction potentials and the dimensionless depletion forces. Taking into account the above-mentioned arguments of calculation, the free energy of the system, the respective partition function $Z_{\tilde{f},\|}\left(L\right)$ of a dilute solution of star polymers with an odd number $\tilde{f}=3,5$ of arms immersed in a slit of two parallel walls at a distance *L* should be normalized on the partition function *Z* of one star polymer with the respective number of arms in the same volume *V* without walls, as was mentioned above. This offers us the possibility to obtain according to Equation (18) the results for the dimensionless depletion interaction potentials $\Theta_{\tilde{f}}^{\left(DD\right)}\left(y\right)$ of a dilute solution of star polymers with three and five arms inside a slit with two repulsive walls, which are, respectively:

(24) $$\Theta_{\tilde{f}=3}^{\left(DD\right)}\left(y\right)\approx -\sqrt{\frac{14}{\pi }}y^{2}\left(\frac{4}{5}e^{-\frac{9}{14}y^{2}}-\frac{36}{7}e^{-\frac{18}{7}y^{2}}+\ldots \right),$$

(25) $$\Theta_{\tilde{f}=5}^{\left(DD\right)}\left(y\right)\approx -\sqrt{\frac{26}{\pi }}y^{2}\left(\frac{2350}{351}e^{-\frac{25}{26}y^{2}}-\frac{25720}{351}e^{-\frac{50}{13}y^{2}}+\ldots \right),$$

where $y=\frac{L}{R_{x}}$.

We can compare the results obtained in Equations (24) and (25) for an odd number of arms with the results previously obtained in [8] for a star polymer with an even number of arms $\tilde{f}=4$.

Taking into account Equation (19) in Section 2, the dimensionless depletion forces $\Gamma_{\tilde{f}}^{\left(DD\right)}\left(y\right)$ for star polymer chains with three and five arms immersed in the slit with D-D b.c. are obtained:

$$\begin{array}{c} \Gamma_{\tilde{f}=3}^{\left(DD\right)}\left(y\right)\approx \sqrt{\frac{14}{\pi }}ye^{-\frac{9}{14}y^{2}}\left(\frac{8}{5}-\frac{36}{35}y^{2}\right)- \end{array}$$

(26) $$\begin{array}{c} \sqrt{\frac{14}{\pi }}ye^{-\frac{18}{7}y^{2}}\left(\frac{72}{7}-\frac{1296}{49}y^{2}\right)+\ldots , \end{array}$$

$$\begin{array}{c} \Gamma_{\tilde{f}=5}^{\left(DD\right)}\left(y\right)\approx \sqrt{\frac{26}{\pi }}ye^{-\frac{25}{26}y^{2}}\left(13.39-12.88y^{2}\right)- \end{array}$$

(27) $$\begin{array}{c} \sqrt{\frac{26}{\pi }}ye^{-\frac{50}{13}y^{2}}\left(146.55-563.66y^{2}\right)+\ldots . \end{array}$$

We can compare the results obtained in Equations (26) and (27) for an odd number of arms with the results for a star polymer with an even number of arms $\tilde{f}=4$, obtained in our previous paper [8].

**Figure 1:**
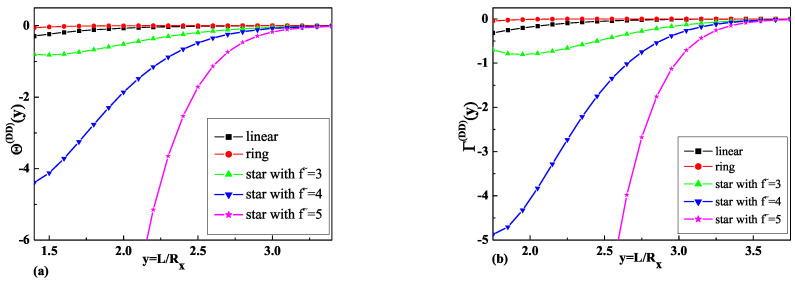
(**a**) The comparison of the obtained results for the dimensionless depletion interaction potentials of star polymer chains with a different number of arms $\tilde{f}$ immersed in a slit with D-D b.c. and the results for linear [36] and ring [8,25] polymer chains. (**b**) The comparison of the obtained results for the dimensionless depletion forces of star polymer chains with a number of arms $\tilde{f}$ immersed in a slit with D-D b.c. and the results for linear [36] and ring [8,25] polymer chains.

The results obtained in Equations (24) and (25) for the dimensionless depletion interaction potentials $\Theta_{\tilde{f}}^{\left(DD\right)}\left(y\right)$, as well as the results obtained in Equations (26) and (27) for the dimensionless depletion forces $\Gamma_{\tilde{f}}^{\left(DD\right)}\left(y\right)$ for ideal star polymer chains with an odd number $\tilde{f}=3,5,$ of arms immersed between two repulsive walls are presented in Figure 1a and Figure 1b, respectively.

Now, we proceed to the case of a dilute solution of ideal star polymers with an odd number $\tilde{f}=3,5$ of arms immersed inside a slit with mixed boundary conditions (D-N b.c.). Following the above mentioned scheme, for the case of the dimensionless depletion interaction potentials, in this case, we obtain the following results:

(28) $$\Theta_{\tilde{f}=3}^{\left(DN\right)}\left(y\right)\approx -\frac{8}{5}y^{2}\sqrt{\frac{14}{\pi }}e^{-\frac{18}{7}y^{2}}+\ldots ,$$

(29) $$\Theta_{\tilde{f}=5}^{\left(DN\right)}\left(y\right)\approx -24.06y^{2}\sqrt{\frac{26}{\pi }}e^{-\frac{50}{13}y^{2}}+\ldots$$

which we can compare with the results for a star polymer with an even number of arms $\tilde{f}=4$, obtained in our previous paper [8].

The dimensionless depletion force $\Gamma_{\tilde{f}}^{\left(DN\right)}\left(y\right)$ for star polymers with three and five arms in the slit with mixed boundary conditions (D-N b.c.) are, respectively,

(30) $$\Gamma_{\tilde{f}=3}^{\left(DN\right)}\left(y\right)\approx \sqrt{\frac{14}{\pi }}ye^{-\frac{18}{7}y^{2}}\left(\frac{16}{5}-\frac{288}{35}y^{2}+\ldots \right),$$

(31) $$\Gamma_{\tilde{f}=5}^{\left(DN\right)}\left(y\right)\approx \sqrt{\frac{26}{\pi }}ye^{-\frac{50}{13}y^{2}}\left(48.12-185.09y^{2}+\ldots \right).$$

**Figure 2:**
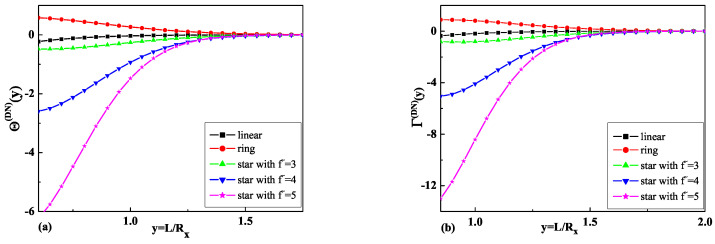
(**a**) The comparison of the obtained results for the dimensionless depletion interaction potentials of star polymer chains with a different number of arms $\tilde{f}$ immersed in a slit with D-N b.c. and the results for linear [36] and ring polymer [8,25] chains. (**b**) The comparison of the obtained results for the dimensionless depletion forces of star polymer chains with a number of arms $\tilde{f}$ immersed in a slit with D-N b.c. and the results for linear [36] and ring polymer [8,25] chains.

The results obtained in Equations (28) and (29) for the dimensionless depletion interaction potentials $\Theta_{\tilde{f}}^{\left(DN\right)}\left(y\right)$, as well as the results obtained in Equations (30) and (31) for the dimensionless depletion forces $\Gamma_{\tilde{f}}^{\left(DN\right)}\left(y\right)$ for ideal star polymer chains with an odd number $\tilde{f}=3,5$ of arms immersed in the slit with mixed boundary conditions (D-N b.c.) are presented in Figure 2a and Figure 2b, respectively.

To understand the influence of polymer chain topology on the depletion interaction potentials and the depletion forces, Figure 1 and Figure 2 also present results for ideal linear [36] and ring polymer chains [25]. As is possible to see from Figure 1b and Figure 2b, the depletion force in the case of D-D b.c. has a greater absolute value than in the case of D-N b.c. for all types of star polymer chains and a greater absolute value than the respective forces for linear and ring polymer chains with $0_{1}$ topology, where the standard notation $C_{p}$ [46] was used. The dimensionless depletion force for star polymers with an odd and even number arms in the case of D-N b.c. demonstrates the opposite behavior to the depletion force for ring polymer chains and is repulsive. As is possible to see from Figure 1b and Figure 2b, the absolute value of the depletion force increases when the number of arms increases.

### 3.2. The Layer Monomer Density for Star-Shaped Polymers

We performed an investigation into the layer monomer densities of star polymers $\rho_{\lambda ,\tilde{f}}\left(\tilde{z}\right)$ defined by:

(32) $$\rho_{\lambda ,\tilde{f}}\left(\tilde{z}\right)d\tilde{z}=\frac{{\left(2R_{g,\tilde{f}}\right)}^{1/\nu }}{N}dN_{\lambda ,\tilde{f}}\left(\tilde{z}\right),$$

where the value $dN_{\lambda ,\tilde{f}}\left(\tilde{z}\right)$ means the number of monomers in the layer between $\tilde{z}$ and $\tilde{z}+d\tilde{z}$, and ν is a Flory critical exponent, as was mentioned in Section 2 above.

In general, the layer monomer densities $\rho_{\lambda ,\tilde{f}}\left(\tilde{z}\right)$ can be obtained from monomer density $\rho_{\tilde{f}}\left(\tilde{r},\tilde{z}\right)$ after integration over the d−1 components parallel to the wall. It should be mentioned that the scaling dimensions of $\rho_{\tilde{f}}\left(\tilde{r},\tilde{z}\right)$ is $l^{1/\nu -d}$ and equals the ordinary dimensions of the quantity:

(33) $$\Psi_{a}\left(\tilde{x}\right)=\frac{{\left(2R_{g}\right)}^{1/\nu }}{2L_{0}}\Phi_{a}^{2}\left(\tilde{x}\right),$$

where $\Phi_{a}^{2}\left(\tilde{x}\right)$ are the insertions of the operators connected with source terms, which are added to the Hamiltonian $H_{st}$ in Equation (7) and appear in the corresponding generation and correlation functions (see [37]).

The layer monomer densities of a single star polymer chain trapped inside a slit of two parallel walls can be obtained by analogy, as was proposed for linear polymer chains in [15], and can be written in the following form:

(34) $$<\rho_{\tilde{f}}\left(\tilde{x}\right)>=\frac{{\mathrm{IL}}_{\mu_{0}^{2}\to L_{0}^{\prime }}\sum_{a=1}^{\tilde{f}}<\Psi_{a}\left(\tilde{x}\right)\cdot {\vec{\phi }}_{a}\left(x\right){\vec{\phi }}_{a}\left(x^{\prime }\right){>}_{ww}}{{\mathrm{IL}}_{\mu_{0}^{2}\to L_{0}^{\prime }}\sum_{a=1}^{\tilde{f}}<{\vec{\phi }}_{a}\left(x\right){\vec{\phi }}_{a}\left(x^{\prime }\right){>}_{ww}}$$

in the limit n→0. The average $<{>}_{ww}$ in Equation (34) denotes a statistical average for a Ginzburg–Landau field theory inside a slit between two walls. The dot in Equation (34) means the usual cumulant average and IL is the inverse Laplace transform $\mu_{0}^{2}\to L_{0}^{\prime }$, where the value $L_{0}^{\prime }$ determines the number of monomers of the corresponding star polymer such that $L_{0}^{\prime }$ equals $R_{g,\tilde{f}}^{2}$ for an ideal star polymer with the respective number of arms in the bulk.

According to the normalization condition: $\int d^{d}\tilde{x}<\rho_{\tilde{f}}\left(\tilde{x}\right)>={\left(2R_{g,\tilde{f}}\right)}^{1/\nu }$ the property:

$$\begin{array}{ccc} \int_{0}^{L}d\tilde{z}\int d^{d-1}\tilde{r}{\mathrm{IL}}_{\mu_{0}^{2}\to L_{0}^{\prime }}\sum_{a=1}^{\tilde{f}}<\Psi_{a}\left(\tilde{r},\tilde{z}\right)\cdot {\vec{\phi }}_{a}\left(x\right){\vec{\phi }}_{a}\left(x^{\prime }\right){>}_{ww} & = & \end{array}$$

(35) $$\begin{array}{c} {\left(2R_{g,\tilde{f}}\right)}^{1/\nu }{\mathrm{IL}}_{\mu_{0}^{2}\to L_{0}^{\prime }}\sum_{a=1}^{\tilde{f}}<{\vec{\phi }}_{a}\left(x\right){\vec{\phi }}_{a}\left(x^{\prime }\right){>}_{ww}, \end{array}$$

takes place.

Near the repulsive wall with Dirichlet b.c. (D b.c.), the short-distance expansion of $\Phi_{a}^{2}$ can be used [15,35,47,48], which in the present case has the form:

(36) $$\Psi_{a}\left(\tilde{r},\tilde{z}\right)\to B{\tilde{z}}^{1/\nu }\frac{{\left[\Phi_{a,⊥}\left(\tilde{r}\right)\right]}^{2}}{2},$$

for distances $l\ll \tilde{z}$. The surface operator $\frac{{\left[\Phi_{a,⊥}\left(\tilde{r}\right)\right]}^{2}}{2}$ with $\Phi_{a,⊥}=\frac{\partial \Phi_{a}\left(\tilde{r},\tilde{z}\right)}{\partial \tilde{z}}{|}_{\tilde{z}=0}$ is the component of the stress tensor perpendicular to the walls. Taking into account the correspondent shift identity [34,35,49] in the case of a slit of two parallel walls situated at a distance *L* from each other, for the layer monomer densities $\rho_{\lambda ,\tilde{f}}\left(\tilde{z}\right)$ of star polymers in accordance with Equations (34) and (36), the universal density force relation can be obtained for the region $l\ll \tilde{z}\ll R_{g,\tilde{f}}$ and can be presented in a form similar to the case of linear polymer chains [15]:

(37) $$<\rho_{\lambda ,\tilde{f}}\left(\tilde{z}\right)>=B{\tilde{z}}^{1/\nu }\frac{f_{\tilde{f}}}{k_{B}T},$$

where

(38) $$\frac{f_{\tilde{f}}}{k_{B}T}=\frac{d}{dL}\ln\left[{\mathrm{IL}}_{\mu_{0}^{2}\to L_{0}^{\prime }}\int d^{d}x\int d^{d}x^{\prime }\sum_{a=1}^{\tilde{f}}<{\vec{\phi }}_{a}\left(x\right){\vec{\phi }}_{a}\left(x^{\prime }\right){>}_{ww}\right]$$

is the force per area that the star polymer exerts on the walls inside the slit.

As is known [15,35], the resulting force per area $\frac{f_{\tilde{f}}}{k_{B}T}$ exerted on the surfaces of a confining slit by the polymer chain has the opposite sign to the depletion force $\Gamma_{\tilde{f}}\left(y\right)$ in Equation (19). The calculation of the force per area that the star polymer exerts on the walls inside the slit $\frac{f_{\tilde{f}}}{k_{B}T}$ in Equation (38) is connected with the calculation of the contribution of $\omega_{\tilde{f}}$ (see Equation (21)) to the total grand canonical free energy $\Omega_{\tilde{f}}$ in Equation (20).

The universal amplitude *B* is identified via scaling relations for the monomer density and force and can be written in the form: $B=\lim_{x\to 0}x^{-1/\nu }X\left(x,y\right)/Y\left(y\right)$, where *X* and *Y* are universal functions.

Taking into account the Derjaguin approximation [43], we performed the calculation of the layer monomer density profiles in the case when we have a dilute polymer solution of star polymers with an odd number $\tilde{f}=3,5$ of arms immersed in a solution of big colloidal particles with different adsorbing or repelling properties in respect to the star polymers, and compared them with the results for an even number of arms with $\tilde{f}=4$, obtained in [8] and the results for linear [36] and ring polymers [8,25]. Moreover, we discussed two cases of immersing a dilute solution of star polymers in confined geometries: (1) between a wall and a big colloidal particle and (2) between two big colloidal particles with D-D b.c. and D-N b.c., respectively.

The Derjaguin approximation [43], which describes the sphere by a superposition of fringes with a local distance from the wall $L\left(r_{\|}\right)=\tilde{a}+r_{\|}^{2}/\left(2R\right)$, can be applied in the case of a spherical mesoscopic colloidal particle with radius *R* much larger than the distance of its closest point $“\tilde{a}”$ to the surface and much larger than the radius of gyration $R_{g,\tilde{f}}$ of the star polymer. Immersing the big spherical colloidal particle in a dilute solution of star polymers confined in a semi-infinite space changes the force exerted on the wall by the value $\Delta f_{\tilde{f}}$, where the index $\tilde{f}$ corresponds to the number of arms in the star polymer. The depletion interaction of the particle with the wall can be obtained as the difference between the forces with and without the particle. The above-mentioned arguments and the universal density force relation in Equation (37) allow us to obtain the expression for the layer monomer density profiles of a dilute solution of star polymers with a different number of arms in a semi-infinite geometry containing a spherical particle of big radius in the form:

(39) $$<\rho_{\lambda ,\tilde{f}}\left(\tilde{z}\right){>}_{wp}=B{\tilde{z}}^{1/\nu }\left(\frac{\Delta f_{\tilde{f}}}{k_{B}T}+n_{B}\right),$$

by analogy, as was proposed for the case of linear polymer chains in [18,19]. Here, $n_{B}=\tilde{N}/V$ is the number density of polymer chains in the bulk solution and $\tilde{N}$ is the total amount of star polymers in the solution. The depletion interaction potential for a dilute solution of star polymers between the particle and the wall can be obtained according to [18,36] in the form:

(40) $$\frac{\phi_{depl,\tilde{f}}\left(\tilde{a}\right)}{n_{B}k_{B}T}=2\pi RR_{x}^{2}\int_{\frac{\tilde{a}}{R_{x}}}^{\infty }dy\Theta_{\tilde{f}}\left(y\right),$$

and it allows us to calculate the contribution to the force per area that the star polymer exerts on the surfaces of the particle and the wall (or on the surfaces of two particles):

(41) $$\Delta f_{\tilde{f}}=d\phi_{depl,\tilde{f}}\left(\tilde{a}\right)/d\tilde{a}.$$

In the case when we consider a dilute solution of star polymers between a wall and a big colloidal particle, the value *R* corresponds to the radius $R=\tilde{R}$ of the particle. In the case when we have two big colloidal particles, we have $R=\frac{R_{1}R_{2}}{R_{1}+R_{2}}$ with $R_{1}\neq R_{2}$. $\Theta_{\tilde{f}}\left(y\right)$ in Equation (40) is the dimensionless scaling function of the free energy of a dilute solution of star polymers confined in a slit, which was obtained in the previous section. Taking into account the above-mentioned arguments, we can write the results for the force $\frac{\Delta f_{\tilde{f}}}{n_{B}k_{B}T}$ in the case of star polymers with an odd number $\tilde{f}=3,5$ of arms and compare them with the results for $\tilde{f}=4$ arms [8]. Thus, in the case when the surface of a big colloidal particle and the surface of the confining wall (or two colloidal particles) are at the D-D b.c., we obtain the following results for the force $\frac{\Delta f_{\tilde{f}}^{\left(DD\right)}}{n_{B}k_{B}T}$ with an odd number of arms:

(42) $$\frac{\Delta f_{\tilde{f}=3}^{\left(DD\right)}}{n_{B}k_{B}T}\approx \sqrt{14\pi }RR_{x}\frac{{\tilde{a}}^{2}}{R_{x}^{2}}\left(\frac{8}{5}e^{-\frac{9}{14}\frac{{\tilde{a}}^{2}}{R_{x}^{2}}}-\frac{72}{7}e^{-\frac{18}{7}\frac{{\tilde{a}}^{2}}{R_{x}^{2}}}\right),$$

(43) $$\begin{array}{ccc} \frac{\Delta f_{\tilde{f}=5}^{\left(DD\right)}}{n_{B}k_{B}T} & \approx & \sqrt{26\pi }RR_{x}\frac{{\tilde{a}}^{2}}{R_{x}^{2}}\left(13.39e^{-\frac{25}{26}\frac{{\tilde{a}}^{2}}{R_{x}^{2}}}-146.55e^{-\frac{50}{13}\frac{{\tilde{a}}^{2}}{R_{x}^{2}}}\right). \end{array}$$

Moreover, in the case of mixed walls with D-N b.c., we obtain for the force $\frac{\Delta f_{\tilde{f}}^{\left(DN\right)}}{n_{B}k_{B}T}$ with an odd number of arms the following results:

(44) $$\frac{\Delta f_{\tilde{f}=3}^{\left(DN\right)}}{n_{B}k_{B}T}\approx \frac{16}{5}\sqrt{14\pi }RR_{x}\frac{{\tilde{a}}^{2}}{R_{x}^{2}}e^{-\frac{18}{7}\frac{{\tilde{a}}^{2}}{R_{x}^{2}}},$$

(45) $$\frac{\Delta f_{\tilde{f}=5}^{\left(DN\right)}}{n_{B}k_{B}T}\approx 48.12\sqrt{26\pi }RR_{x}\frac{{\tilde{a}}^{2}}{R_{x}^{2}}e^{-\frac{50}{13}\frac{{\tilde{a}}^{2}}{R_{x}^{2}}}.$$

Taking into account Equation (40) and the results presented in Figure 1a and Figure 2a for the dimensionless depletion interaction potentials, we can see that the absolute value of the dimensionless depletion interaction potentials of star polymers increase when the number of arms of the star polymers increases. The absolute value of the dimensionless depletion interaction potentials in the case when the particle and the wall are repulsive in respect to polymer (the case of D-D b.c.) is definitely greater than in the case when the particle and the wall have mixed boundary conditions (the case of D-N b.c.). Such behavior is observed for all numbers of arms in star polymers.

**Figure 3:**
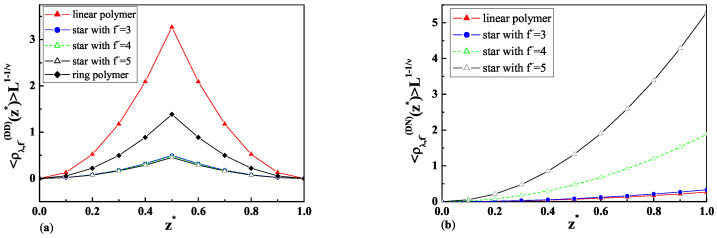
(**a**) The layer monomer density profiles of a dilute solution of star polymers with a different number of legs $\tilde{f}$ immersed between a particle and a wall in the case of D-D b.c. with L=50[l], R=20[l], and $\tilde{a}=0.015\left[l\right]$. (**b**) The layer monomer density profiles of a dilute solution of star polymers with a different number of legs $\tilde{f}$ immersed between a particle and a wall in the case of D-N b.c. with L=50[l], R=10[l], and $\tilde{a}=1.3\left[l\right]$.

The results of calculations for the layer monomer density profiles of a dilute solution of star polymers with a different number of legs $\tilde{f}$ immersed between a particle and a wall in the case of D-D b.c. and D-N b.c. according to Equation (39) and Equations (42) and (43) and Equations (44) and (45) are presented in Figure 3a and Figure 3b, respectively.

As is possible to see from Equations (39)–(41) and (13), the layer monomer densities depend not only on $R_{g,\tilde{f}}$, but also on the size of the mesoscopic particle *R* and its distance $\tilde{z}$ from the wall. The results of calculations for the layer monomer density profiles $<\rho_{\lambda ,\tilde{f}}\left(\tilde{z}\right){>}_{wp}$ (Equations (39)–(41)) of a dilute solution of star polymers with a different number of arms in a semi-infinite geometry containing a particle of big radius in the case of D-D b.c. (see Equations (42) and (43)), and D-N b.c. (see Equations (44) and (45)) are presented in Figure 3a and Figure 3b, respectively. As is possible to see from Figure 3a, the maximum of the layer monomer density profiles is in the middle of the distance between the particle and the wall in the case of D-D b.c. For distances $z^{*}=\tilde{z}/L$ bigger than half of the distance between particle and wall ($z^{*}>0.5$), the layer monomer density profiles symmetrically decrease to zero. Moreover, increasing the star polymer complexity of the structure leads to a reduction in the layer monomer density profiles value at the D-D b.c. In the case of D-N b.c., the maximum of the layer monomer density profiles is observed at the wall where the adsorption threshold takes place (see Figure 3b). In this case, star polymers with a more complicated topological structure have higher values of the layer monomer density profiles at the wall where the adsorption threshold takes place.

We can see that in the case when we have two particles of the same size, the respective contribution to the layer monomer density profiles $<\rho_{\lambda ,\tilde{f}}\left(\tilde{z}\right){>}_{wp}$ from immersing the particles becomes twice smaller than in the case when we have one particle near the wall.

### 3.3. Results of Molecular Dynamic Simulations of Linear, Ring, and Star-Shaped Polymers in a Slit

We performed molecular dynamics simulations of star polymers with an odd number of arms modifying the software that we wrote in C++ previously [8]. Polymers with three and five arms were built of 901 and 1501 monomers, respectively (central monomer and an appropriate number of arms, each containing 300 particles). The attractive and repulsive interactions of the neighboring monomers in a polymer chain were modeled using the finite extensible nonlinear elastic (FENE) and the Weeks–Chandler–Andersen (WCA) potentials, respectively. Potential was chosen to preserve the topologies of polymers, as it has one minimum and for r→∞ potential U(r)→∞. Non-neighboring monomers interacted by the 12–6 Lennard–Jones potential. We used the Verlet integration algorithm with Δt=0.005. In order to keep the temperature constant in the NVT ensemble, we used the velocity scaling thermostat, which was applied during the integration scheme. We set temperature *T* to 1.

The monomer–wall interaction with two walls at a distance *L* from one to another is given by the 9–3 Lennard–Jones potential with a cut-off [50]:

(46) $$U_{LJ_{9-3}}\left(r\right)=\frac{3\sqrt{3}}{2}\epsilon \left[{\left(\frac{\sigma }{r}\right)}^{9}-{\left(\frac{\sigma }{r}\right)}^{3}\right].$$

We assumed ϵ=1 and σ=1. The cut-off of the monomer–monomer potential depends on the boundary conditions. The potential cut-off was set to either $R_{cut-off}=3^{1/6}$ for a repulsive wall and $R_{cut-off}=10$ for an attractive one.

As was mentioned above, the monomer–monomer potential is composed of FENE (attractive part) and WCA (repulsive part).

The FENE potential [51] is given by:

(47) $$U_{FENE}\left(r\right)=\left\{\begin{array}{cc} -\epsilon & \mathrm{if}\;r<2^{1/6}\sigma ,\\ 4\epsilon \left[{\left(\frac{\sigma }{r}\right)}^{12}-{\left(\frac{\sigma }{r}\right)}^{6}\right] & \mathrm{if}\;r\geq 2^{1/6}\sigma . \end{array}\right.$$

We assumed ϵ=1 and σ=1. For the repulsive part, we used:

(48) $$U_{WCA}\left(r\right)=\left\{\begin{array}{cc} -\frac{1}{2}kR_{0}^{2}\ln\left[1-{\left(\frac{r}{R_{0}}\right)}^{2}\right] & \mathrm{if}\;r<R_{0},\\ +\infty & \mathrm{if}\;r\geq R_{0}. \end{array}\right.$$

We assumed k=30 and $R_{0}=3/2$. The cut-off of the repulsive part of the monomer–monomer potential is equal to $R_{cut-off}=2^{1/6}$.

As the first task, the program with no boundary conditions was run for each polymer shape 10 times to determine the radius of gyration $R_{g,\tilde{f}}$. All simulations were initially equilibrated for t=500 and then all data were collected for t=3000. As the result, we obtained $R_{g,\tilde{f}=3}=30.69$ and $R_{g,\tilde{f}=5}=28.51$ for three- and five-arm star polymers, respectively. The results are similar to those for four-arm stars obtained in [8]; however, polymers with more arms have a smaller radius of gyration. This is due to the excluded volume effect at the joint of the arms and the higher monomer density closer to the core of a molecule. Moreover, we performed the calculation of the ratio $R_{g⊥}/R_{g\|}$ of the perpendicular to the surfaces $R_{g⊥}$ and the parallel to the surfaces $R_{g\|}$ contribution to the radius of gyration. The results of the calculation of the ratio $R_{g⊥}/R_{g\|}$ for a dilute solution of star polymers with an odd number $\tilde{f}=3,5$ of arms for the case of a narrow slit with $L=0.5R_{g,\tilde{f}}$ and the case of a wide slit with $L=2.0R_{g,\tilde{f}}$ for different boundary conditions N-N b.c., N-D b.c., and D-D b.c. are presented in Table 1.

Knowing the $R_{g,\tilde{f}}$, we performed simulations with various boundary conditions (two repulsive walls, two attractive walls, and one attractive and one repulsive wall) for the case of a wide slit with $L=2R_{g,\tilde{f}}$ and a narrow slit with $L=0.5R_{g,\tilde{f}}$. We performed the calculation of the monomer density profiles, which were normalized as follows: the separations *z* of the walls were normalized to 1 and monomer densities as $\int_{0}^{1}\rho \left(z\right)=\frac{R_{g,\tilde{f}}}{L}$. Simulations of polymers with an odd number of arms were more difficult than the previous ones for an even number $\tilde{f}=4$ of arms [8], because in the case of two attractive walls, arms of an uneven number always stick to the walls. Therefore, in this case, density profiles were symmetrized in the following way: ($\rho_{new}\left(z\right)=\frac{\rho (z)+\rho (1-z)}{2}$).

**Figure 4:**
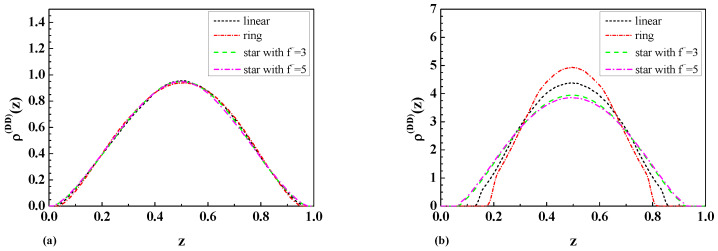
The comparison of the monomer density profiles ρ(z) of linear [8] and star-shaped polymers with number of arms $\tilde{f}$ between two repulsive walls with a separation of (**a**) $L=2R_{g,\tilde{f}}$ (wide slit) and (**b**) $L=R_{g,\tilde{f}}/2$ (narrow slit).

In the case of two repulsive walls in a wide slit region with separation $L=2R_{g,\tilde{f}}$, we observed that the monomer density profiles for star polymers are higher than the respective results for linear and ring polymers in the middle of the slit and near the walls, as is possible to see in Figure 4a. As one can see from Figure 4b, the situation looks completely different in the case of a narrow slit. In this case, the monomer density profiles for ring polymers (see [8]) are higher than the respective results for linear and star polymers with a different number of arms in the middle of the slit. Such behavior is connected with the different topologies of the polymers, as well as their corresponding values $R_{g,\tilde{f}}$ of the radius of gyration. It should be mentioned that the behavior of the monomer density profiles near the walls is completely the opposite.

**Figure 5:**
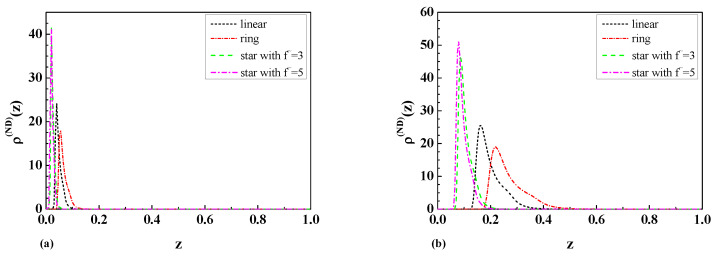
The comparison of the monomer density profiles ρ(z) of linear [8] and star-shaped polymers with number of arms $\tilde{f}$ between one repulsive and one attractive wall with a separation of (**a**) $L=2R_{g,\tilde{f}}$ (wide slit) and (**b**) $L=R_{g,\tilde{f}}/2$ (narrow slit).

In the case of one attractive and one repulsive wall (Figure 5a,b), we observed that the monomer density profiles for star polymers are higher than the corresponding results for linear and ring polymers. Furthermore, the maxima of peaks for the above mentioned cases are shifted for the case of wide and narrow slits. As is possible to see from Figure 5a,b, in the case of a wide slit, the polymers are not influenced by the presence of the repulsive wall. The situation looks different in the case of a narrow slit, where the positions and shapes of peaks are shifted when compared to the case of a wide slit.

**Figure 6:**
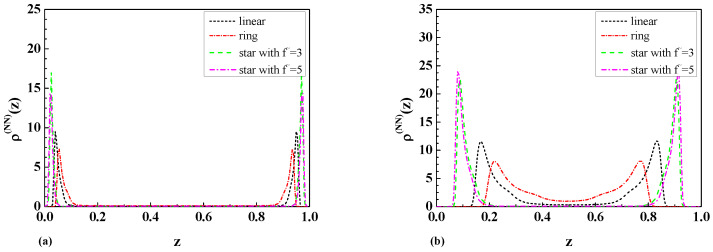
The comparison of the monomer density profiles ρ(z) of linear [8] and star-shaped polymers with number of arms $\tilde{f}$ between two attractive walls with a separation of (**a**) $L=2R_{g,\tilde{f}}$ (wide slit) and (**b**) $L=R_{g,\tilde{f}}/2$ (narrow slit).

Figure 6a,b present the result for the case of two attractive walls. The resulting monomer density profiles indicate that the polymer tends to stay near the attractive walls. The biggest difference is observed in the case of a narrow slit where a non-zero monomer density is observed in the middle of the slit, especially in the case of ring polymers.

As is possible to see from Figure 4a–Figure 6b, the topological and entropic effects play a crucial role in the monomer density profiles near the walls. The obtained molecular dynamic simulation results for the monomer density profiles for the case of two repulsive walls (see Figure 4a,b) and the case of one repulsive and one attractive wall (see Figure 5a,b) qualitatively coincide with the analytical results for the layer monomer density profiles presented in Figure 3a and Figure 3b, respectively. The difference in absolute value is connected with the fact that the analytical calculations in Figure 3a,b were performed for a dilute solution of star polymers with a number of monomers N→∞ immersed in a solution of big colloidal particles with different adsorbing or repelling properties in respect to the star polymers, but the numerical calculations were performed for the case of a dilute polymer solution of star polymers with a fixed number of monomers immersed inside a slit.

## 4. Discussion and Conclusions

The present paper is devoted to the analytical investigation of a dilute solution of ideal star polymers with an odd number $\tilde{f}=3,5$ of arms immersed in a Θ-solvent and confined in a slit of two parallel walls as well as in a solution of big spherical colloidal particles which is characterized by different adsorbing and repelling properties in respect to the polymers.

The dimensionless depletion interaction potentials and the dimensionless depletion forces for a dilute solution of ideal star polymers with an odd number $\tilde{f}=3,5$ of arms in a Θ-solvent confined in a slit of two parallel walls with repulsive surfaces and for the case of one repulsive and inert surface were obtained analytically and compared with the results for a star polymer with an even number of arms $\tilde{f}=4$, obtained previously in [8]. The obtained results indicate that the depletion force in both cases for a dilute solution of star polymers is attractive, but greater than the respective forces for linear and ring polymers. It should be noticed that the depletion force in the case of walls with mixed boundary conditions is definitely smaller than in the case of two repulsive surfaces.

Taking into consideration the Derjaguin approximation, the dimensionless layer monomer density profiles of a dilute solution of star polymers with an odd number $\tilde{f}$ of arms confined in a half space containing the mesoscopic spherical colloidal particle of big radius *R* (or two big colloidal particles with $R_{1}\neq R_{2}$) for the case of D-N b.c. and D-D b.c. were obtained. From Equations (39)–(41) and the results presented in Equations (42)–(45), we can see that the layer monomer density depends on the radius of gyration of the star polymers (see Equation (13)), the size of the mesoscopic colloidal particle *R*, and the distance between the wall and the particle or between two particles.

As mentioned above, we obtained the numerical results for the monomer density profiles of a dilute solution of star polymers with the EVI in a good solvent confined in a slit of two repulsive walls (see Figure 4a,b) and one repulsive and one attractive wall (see Figure 5a,b). The above-mentioned results, obtained in Section 3.3, qualitatively coincide with the analytical results for the layer monomer density profiles obtained in Section 3.1 and Section 3.2 and presented in Figure 3a and Figure 3b, respectively. The difference in absolute value is connected with the fact that the analytical calculations in Section 3.1 and Section 3.2 were carried out for a dilute solution of star polymers with a number of monomers N→∞ immersed in a solution of big colloidal particles with different adsorbing or repelling properties in respect to the star polymers, and the numerical calculations in Section 3.3 were performed for the case of a dilute polymer solution of star polymers with a fixed number of monomers immersed inside a slit.

We come to the conclusion that a more complicated topological structure of star polymers leads to the reduction in the layer monomer density profiles in the vicinity of two repulsive walls, which corresponds to the case of D-D b.c. But an increase in star polymer topological complexity in the case of mixed walls leads to an increase in the layer monomer densities at the adsorbing surface (see Figure 3a,b).

The obtained analytical and numerical results indicate that a dilute solution of star polymers with a different number $\tilde{f}$ of arms can be used for the production of new-generation functional materials because the behavior of these solutions depends on the topology of polymers, as well as on the nature and geometry of confined surfaces. These properties of a dilute solution of star polymers with a different number of arms can find very broad practical application in nano-technology, biotechnology, and medicine for drug and gene transmission, as well as in tissue engineering.

## Figures and Tables

**Table 1:** The value of $R_{g⊥}/R_{g\|}$ for a dilute solution of star polymers with a different number $\tilde{f}=3,5$ of arms for the case of a narrow slit with $L=0.5R_{g,\tilde{f}}$ and the case of a wide slit with $L=2.0R_{g,\tilde{f}}$ for different cases of boundary conditions: N-D b.c. and D-D b.c.

L	b.c.	$\tilde{f}=3$	$\tilde{f}=5$
$0.5R_{g}$	ND	0.007	0.007
$0.5R_{g}$	DD	0.082	0.076
$2.0R_{g}$	ND	0.008	0.010
$2.0R_{g}$	DD	0.341	0.326

## Data Availability

Data are contained within the article.
